# Supplementary material for: In-sensor human gait analysis with machine learning in a wearable microfabricated accelerometer
Source: Commun Eng. 2024 Mar 16;3:48. doi: 10.1038/s44172-024-00193-5 (PMC10955877; doi:10.1038/s44172-024-00193-5)
Supplement: Supplementary file 2 — Supplementary Information [file 44172_2024_193_MOESM2_ESM.pdf]

In-sensor human gait analysis with machine learning in a wearable microfabricated accelerometer: Supplementary Notes

Guillaume Dion[1], Albert Tessier-Poirier[1], Laurent Chiasson-Poirier[1], Jean-François Morissette[1], Guillaume Brassard[1], Anthony Haman[1], Katia Turcot[2] and Julien Sylvestre[1 *]

[1]Institut Interdisciplinaire d'Innovation Technologique, Université de Sherbrooke, Sherbrooke, Canada

[2]Interdisciplinary Center for Research in Rehabilitation and Social Integration (Cirris), Department of Kinesiology, Faculty of Medicine, Université Laval, Québec, Canada

[*]julien.sylvestre@usherbrooke.ca

9 February 2024

Supplementary Note 1 MEMS characterization

The response of the mechanical structures in the MEMS device to electrical and acceleration signals was characterized, in order to narrow down the range of possible operating parameters to explore in the optimization phase (see section Supplementary Note 5). Fig. 2a of the main text shows the response of the beam to frequency sweeps of the drive voltage signal, with the frequency increasing during the measurements, for different drive signal amplitudes. The oscillation amplitude of the beam was measured using the functional block shown in Supplementary Figure 2 (measurement, red), but instead of being digitized by the microcontroller, the output of this circuit was fed to a National Instruments PCIe 6374 16-bit data acquisition (DAQ) card, to sample the envelope of the oscillation signal at 10 kHz. Each frequency sweep lasted 20 ms and was repeated ten times for the same drive signal amplitude, such that the curves shown in fig. 2a are the result of ten averages. No external acceleration was applied during these measurements. Using the same experimental setup as for the frequency sweeps, sweeping the driving signal amplitude (from low to high values) at fixed forcing frequencies from 493.4 kHz to 497.8 kHz in steps of 0.4 kHz produced the twelve topmost curves shown in fig. 2b. In order to show the response for a wider range of frequencies, the eight bottommost curves of fig. 2b (from 498.2 kHz to 501.0 kHz) were extracted from the frequency response data (for decreasing frequency sweeps), since the response to drive amplitude sweeps was not measured for forcing frequencies over 497.8 kHz, for which pull-in of the inertial mass happened before the "jump" in oscillation amplitude

could be observed.

For driving voltage amplitudes over 110 V (outside of the range displayed in figures 2a and 2b), there is a drive frequency within the resonance peak of the clamped beam where the inertial mass, which is polarized at the driving voltage, electrostatically pulls in, in plane and towards the beam, until it rests on the stoppers attached to the anchors. This is due to a constant attractive term (in the parallel plates approximation) of $\varepsilon_0 S V_0^2/4d^2$ in the electrostatic force which is proportional to the square of the driving voltage amplitude, that overcomes the mechanical restoring force. With a capacitor surface $S = 130\ \mu\text{m} \times 50\ \mu\text{m}$, a driving voltage amplitude $V_0 = 110$ V, a fixed gap $d = 8\ \mu$m and ε_0 the vacuum permittivity, the calculated constant electrostatic force is 2.7 μN. With the inertial mass collapsed on its stoppers after pull-in, the gap is reduced to 3 μm and the electrostatic force is 19.3 μN, larger than the calculated restoring force of 17 μN (using an inertial mass spring constant of 3.4 N/m). By reducing the air gap between the beam and inertial mass (which acts as the driving electrode), this pull-in reduces the quality factor of the beam due to increased squeeze-film damping, and sets the upper limit of possible driving voltages since a pulled-in inertial mass cannot be used to sense accelerations.

The acceleration sensitivity of the inertial mass (fig. 2d) was characterized by fixing the beam driving voltage amplitude and frequency at 60 V, 246.5 kHz, and sweeping an acceleration signal amplitude and frequency. For this purpose, the MEMS device was installed on an electrodynamic shaker (SinoCeramics JZK-2) powered by a The Modal Shop 2100E21 amplifier and controlled by a Stanford Research Systems SIM960 PID with its input signal set by the National Instruments PCIe 6374 DAQ, using an Analog Devices ADXL1002z accelerometer board installed on the shaker table as a reference. The PID target for these acceleration sweeps consisted of successive Tukey windowed sinusoids of increasing frequency, from 40 Hz to 1.1 kHz, in 400 frequency steps distributed evenly on a logarithmic scale.

As a result of the sinusoidal displacement of the inertial mass, the oscillation amplitude of the beam was modulated at the same frequency. This latter oscillation amplitude, sampled by the DAQ at 20 kS/s, was then measured by averaging the magnitude of its Hilbert transform over all samples contained in the flat section of the Tukey window (taper parameter $r = 0.1$). This windowing procedure was used to mitigate spurious high frequency signals which were otherwise introduced at the beginning of the acceleration signals, when the acceleration frequency was changed. Since the acceleration response of our electrodynamic shaker system

was not perfectly flat and presented peaks between 300 and 500 Hz, the measured MEMS response was normalized, at each measurement frequency, by the reference acceleration signal amplitude (also obtained through the Hilbert transform) produced by the ADXL 1002z accelerometer and measured by the DAQ, to yield the sensitivity in units of volts of beam oscillation signal per g of applied acceleration. While it helped smooth the curves, this normalization was not perfect as the reference accelerometer and our MEMS device were not mounted at exactly the same point on the shaker table, such that there could be small relative displacements between the two (*e.g.,* due to vibration modes of the shaker table or imperfect mounting on the latter).

The phase of the inertial mass oscillations relative to that of the acceleration signal (fig. 2d, red) was measured by converting the lag (index of the maximum of the cross-correlation between the reference acceleration and the beam oscillation envelope signal) into a phase shift using the nominal acceleration signal frequency and the sample rate.

Supplementary Note 2 Frequency content of gait accelerations

Supplementary Figure 1 shows the power spectral density of the acceleration signal measured by the reference accelerometer in the axis of the top of the shoe (see fig. 1a), with each frequency bin averaged over all participants and gait patterns, for the 0.63 m/s subset of the dataset. The first peak is centered around 0.7 Hz, which corresponds to the average frequency at which steps are taken with the left foot. Most of the information is carried in a 10 Hz bandwidth. Since the sensor was installed on top of the foot, with a slight angle with respect to the floor, this setting is comparable to the one reported in reference 1, according to which 99% of the power of the normal gait vertical force is contained below 15 Hz.

Supplementary Note 3 MEMS system block diagram

The wearable MEMS prototype was assembled from 4 interconnected modules and a battery, as illustrated in Supplementary Figure 2. The oscillation amplitude of the beam was electrostatically coupled to the acceleration-induced displacements of the proof mass by polarizing the latter through a resonant high-voltage

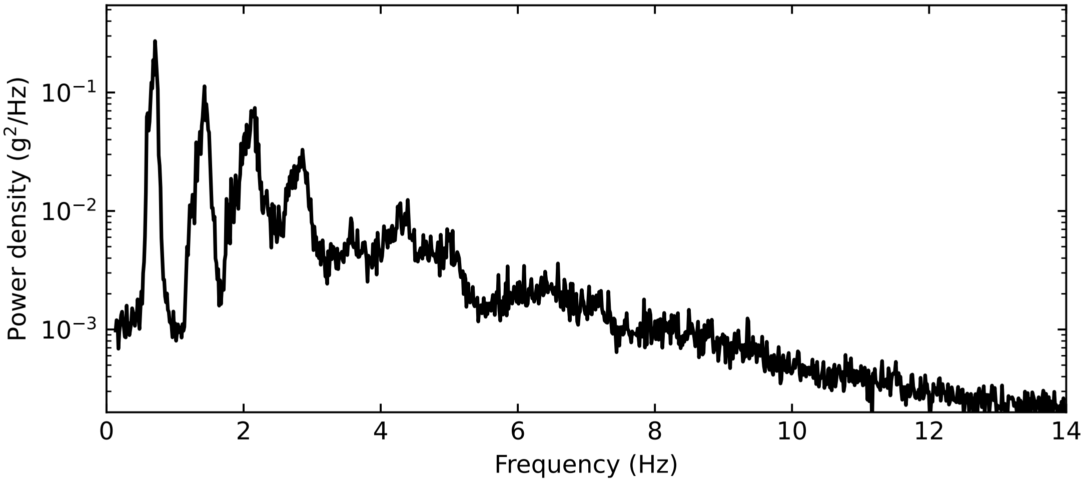

Supplementary Figure 1 | Power spectral density of the acceleration signals sampled by the ADXL326 reference accelerometer, averaged over all four walking patterns and all participants, for a single walking speed of 0.63 m/s.

double-tuned transformer amplifier module composed of two impedance-adapted series transformers (TX) driven by the two phases of a differential output preamplifier (preamp). The high-voltage amplifier could output signals up to 120 V in amplitude at 200 kHz to 300 kHz, which allowed the driving of the doubly clamped silicon beam into its nonlinear regime.

With the resistors of the wheatstone bridge adjusted to correct for the imbalance between the two piezoresistive strain gauge connections, which ensured proper cancellation of the drive signal feedthrough at the differential amplifier stage (diff. amp in Supplementary Figure 2), the instantaneous oscillation signal of the beam was bandpass filtered in a 160 kHz band around its natural frequency before being demodulated by a diode envelope detector (demod in Supplementary Figure 2), amplified and vertically shifted in order to be sampled by the analog-to-digital converter (ADC) of the microcontroller.

The ATSAM4S microcontroller was used to signal the detected walking pattern to the user by enabling a light-emitting diode (LED). It was also used to log and communicate data wirelessly through an external Feather M0 module interfaced by a serial peripheral interface (SPI) using the microcontroller programmable input/output (PIO), although this feature could be disabled to preserve battery life once the system had been trained. Finally, the microcontroller implemented the output layer of the reservoir computer and its feedback

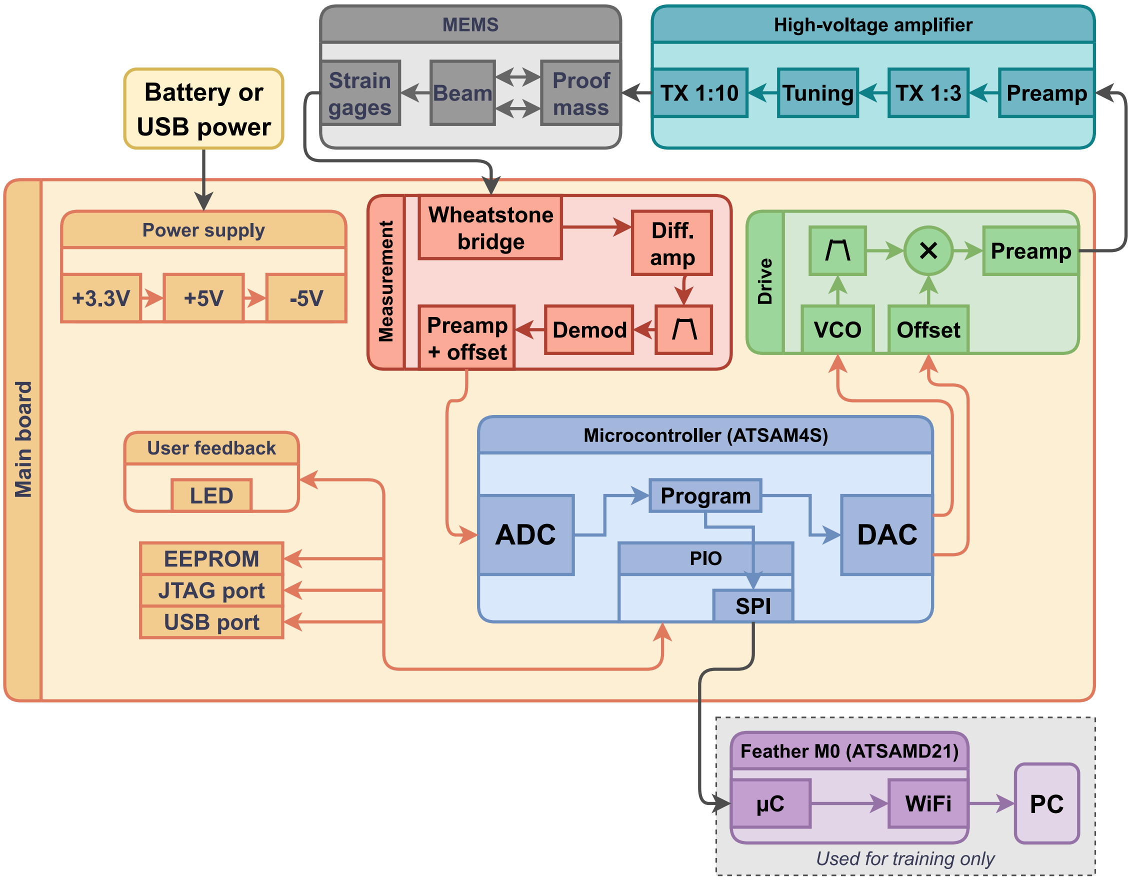

Supplementary Figure 2 | The wearable MEMS system block diagram. The main board (orange) implements the digital logic, analog signal processing and power management blocks, while external modules add functionalities such as WiFi communication (purple), battery power (yellow), MEMS sensing and computing (gray), and drive voltage amplification (green). ADC: analog-to-digital converter; DAC: digital-to-analog converter; Demod: demodulation; Diff. amp: differential amplifier; EEPROM: electrically erasable programmable read-only memory; JTAG: joint test action group interface; LED: light-emitting diode; MEMS: micro-electromechanical system; USB: universal serial bus; PC: personal computer; PIO: programmable input/output; Preamp: preamplifier; SPI: serial peripheral interface; TX: transformer; μC: microcontroller; $\sqcap$: bandpass filter; $\times$: multiplier.

loop. The delayed feedback signal was obtained by delaying the output of the leaky integrator (described in section Training the MEMS system) by 100 samples and scaling it by the feedback gain κ (see Supplementary Table 1). This signal was superposed to the random binary mask signal and the result was output by the microcontroller onboard digital-to-analog converter (DAC) at 14285 Hz.

A voltage-controlled oscillator (VCO) generated a 50% duty square voltage signal at a frequency chosen between 200 kHz and 300 kHz by the microcontroller. This signal was bandpass filtered in a bandwidth of 100 kHz around 250 kHz and was multiplied by the vertically shifted feedback and mask signal, which yielded the amplitude-modulated beam drive signal ready to be amplified by the high-voltage module, thus closing the in-sensor computing loop.

Supplementary Note 4 System integration

Supplementary Figure 3 shows the wearable MEMS system installed on the left shoe for the demonstration of real-time gait analysis (see Supplementary Informations for a video). The MEMS device (microfabricated chip and PCB chip carrier) was placed outside of the main 3D-printed enclosure and it was shielded with metal foil to reduce the amount of electromagnetic and thermal interferences with the main electronic board. It is expected that a MEMS chip packaged using standard industrial processes (including full encapsulation and an integrated metallic lid) would be far less sensitive and could be placed within the main enclosure.

Supplementary Note 5 MEMS system hyperparameters

The hyperparameters of the MEMS system that could be adjusted were: the driving voltage frequency (f_d) and amplitude at the two levels of the binary mask (V_1 and V_2), the delayed feedback gain (κ), the leaking rate (α), the number of virtual nodes (N), the regularization parameter (β) and the virtual node temporal separation (θ). In order to facilitate hyperparameter optimization, the dimensionality of the search space was reduced by using fixed values of $N = 100$ virtual nodes and $\beta = 1 \times 10^{-6}$ for both the MEMS and the ESN systems, and by using a virtual node separation of $\theta = 70$ μs that yields a delay of $N\theta = 7$ ms for the feedback loop in the MEMS system. These parameter values were selected for their general compatibility

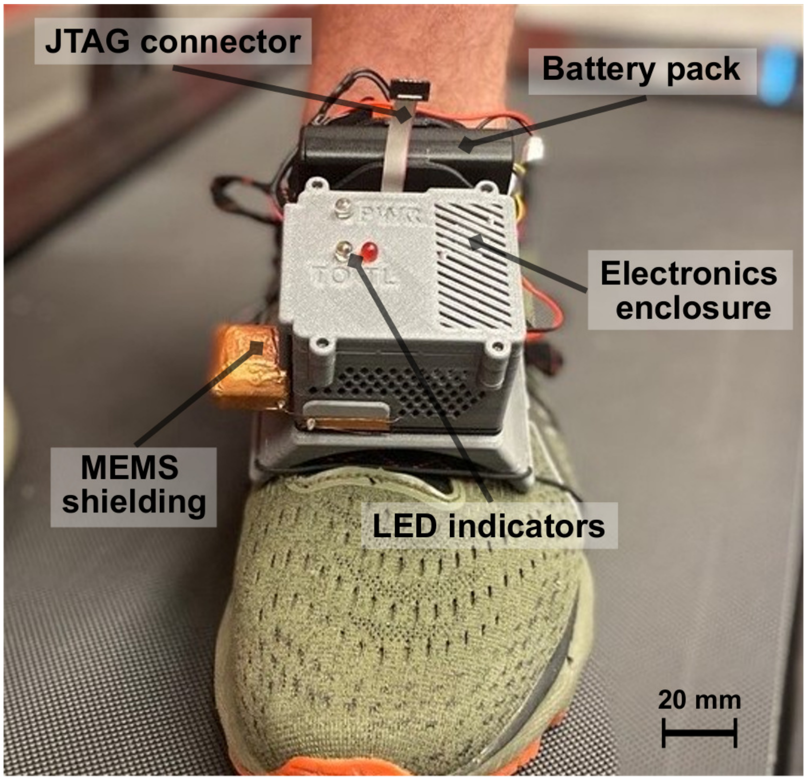

Supplementary Figure 3 | Picture of the wearable real-time system installed on the left shoe. MEMS: micro-electromechanical system; JTAG: joint test action group interface; LED: light-emitting diode.

with the hardware limitations and the timescales of the gait classification task, and from experience with other biomechanical tasks. The other hyperparameters were adjusted with one subject (who was not part of the dataset used in this study) walking on the treadmill at 0.54 m/s, using a bootstrapping approach to estimate the expected value and standard deviation of the mean ROC AUC for each hyperparameter set with a relatively small amount of data.

A segmentation of the recordings by gait cycle was required for the bootstrapping tests, in order to resample complete gait cycles. This segmentation was performed based on the Teager-Kaiser energy operator (TKEO),[3] which outputs the energy signal of the acceleration recordings. The convolution of the low pass filtered TKEO energy signal with a 50 points ramp yielded a signal which peaked at the beginning of the swing phase of each gait cycle, when toes lifted from the ground. This peak, detected with a peak finder, was used to mark the start of each gait cycle. Segmented cycles with a duration under or over 2 standard deviations of the mean cycle duration were discarded.

For each investigated hyperparameter set, the subject walked in the N, TO and TL patterns for 40 cycles each. From the valid segments of the 30 first cycles, 30 cycles were resampled with replacement and used as training data, while 10 cycles were resampled with replacement from the last 10 cycles and used as validation data. This procedure was repeated 150 times in order to estimate the mean and variance of the ROC AUC for a given hyperparameter set. This whole procedure was repeated for 11 random hyperparameter sets which explored different random values of the drive voltage levels V_1 and V_2, feedback gain κ and leaking rate α. The beam driving signal frequency was fixed at 249.4 kHz based on the beam characterization curves. This frequency was chosen to be within the broad resonance peak seen in fig. 2a and to be above the linear resonant frequency (frequency of maximum oscillation amplitude at the lowest forcing amplitude) of 492.5 kHz, to ensure the existence of a driving voltage around which the amplitude response (fig. 2b) was sufficiently nonlinear but without hysteresis. Due to its Duffing nonlinearity and the resulting multi-stability of its dynamics, the oscillation amplitude of our MEMS beam can exhibit jumps (up and down) and hysteresis.[2] For the results presented in this paper, we chose a combination of drive amplitude and drive frequency to operate the MEMS beam in a nonlinear regime without hysteresis. The ESN demonstrated in this paper also used an hyperbolic tangent activation function, that was nonlinear and without hysteresis. The beam driving signal frequency was not systematically optimized, as the aforementioned choice gave satisfactory results,

but further optimization could lead to better performance. As an example, figure 11 from reference 2 shows that, for a similar system performing a different task, adjusting the drive frequency becomes increasingly important in order to retain good performance as the difficulty of the task is increased. The bounds for the random selection of the other hyperparameters were set by manually exploring the hyperparameter space while looking at the reservoir activations, and keeping only the range of parameters for which the activations were correlated with the walking accelerations. Finally, the set of hyperparameters associated with the highest average combined ROC AUC (averaged over all bootstraps and classifiers) was selected.

Since walking speeds higher than the 0.54 m/s adopted for the optimization phase could occasionally cause a pull-in of the inertial mass due to higher acceleration peaks at these speeds, the values of V_1, V_2 and κ were slightly lowered from their optimized values in order to increase stability for a wide range of gaits (from participants having different biomechanical attributes walking at different paces). The resulting lower voltage difference between the inertial mass and oscillating beam reduced the average attractive force between the two mechanical structures such that the allowable range of motion of the inertial mass, and thus the input acceleration limit, was increased.

The optimized hyperparameters are listed in Supplementary Table 1. The hyperparameters for the 'reference system' were used for all the results presented in figures 1 and 3 of this paper. For the demonstration of the wearable system in inference mode in real-time, most of the hyperparameters were kept identical to those of the reference system (see right column of Supplementary Table 1), except for slightly different driving voltage amplitudes V_1 and V_2, and for the feedback gain κ which was set to 0.05. These parameters had to be readjusted in order to accommodate the few differences between the two systems: ±10 V analog signal rails and 16-bit ADC codes converted to (floating point) voltages for the reference system interfaced with the PC, instead of signals limited to ±5 V on the real-time system, with unconverted 12-bit ADC integers used as the virtual node activations.

Supplementary Note 6 ESN hyperparameters

The ESN hyperparameters are given in Supplementary Table 2.

	Reference system	Real-time demonstrator
f_d	249.4 kHz	227.9 kHz
κ	0.7	0.05
α	0.016	0.016
V_1	77.0 V	78 V
V_2	105.6 V	91 V
N	100	100
θ	70 μs	70 μs
β	1×10^{-6} V^2	1×10^{-6} V^2

Table Supplementary Table 1 | Hyperparameter values for the MEMS system.

reservoir size N	100
regularization parameter β	1×10^{-6}
leaking rate α	0.1053
$\boldsymbol{W}_{\text{in}}$ input scaling	2.3
$\boldsymbol{W}_{\text{in}}$ bias scaling	2.9106
sparsity probability for $\boldsymbol{W}_{\text{in}}$	0.59
spectral radius ρ of $\boldsymbol{W}_{\text{r}}$	0.7471
sparsity probability for $\boldsymbol{W}_{\text{r}}$	0.79

Table Supplementary Table 2 | Hyperparameter values for the ESN.

Supplementary Note 7 Power consumption and design optimization

The estimated power consumption of each subcircuit (corresponding to the elements inside each block of Supplementary Figure 2) of the current version of the MEMS wearable system are given in Supplementary Table 3. These values were obtained using the methodology described in the Methods section Power consumption. The power dissipated by the MEMS beam oscillator due to mechanical damping can be approximated, in the case of light damping ($Q \gg 1$), by

$$P_{\text{beam}} \simeq \frac{2\pi f_0 k A^2}{2Q}, \tag{5}$$

where f_0 is the beam oscillation frequency, Q is its quality factor, and $A \sim 1$ μm is its oscillation amplitude. This power is only ~1 μW for the dimensions of the beam (spring constant $k \simeq 128$ N/m), and it is not shown in Supplementary Table 3 since it is negligible compared to the other sources of dissipation. The dissipation in the piezoresistive strain gauges, however, is taken into account through the wheatstone bridge bias (first line of Supplementary Table 3).

Subsystem	Subcircuit	Active device	V_{out} (V_{rms})	Z_{load} (Ω)	I_{load} (mA_{rms})	P_{load} (mW)	$I_{quiescent}$ (mA_{rms})	$P_{quiescent}$ (mW)	P_{total} (mW)	V_{supply} (V) 3.3	5	-5	Power output (mW)	Efficiency (%)
Measurement	Wheatstone bridge	MIC7111YM5	4.00	8.0E+02	5.000	25.000	0.020	0.100	25.100		✕			
	Differential amplifier	INA849DR	0.16	1.1E+04	0.015	0.073	6.000	60.000	60.073		✕	✕		
	Bandpass filter	LM6154	0.12	4.2E+03	0.029	0.143	1.400	14.000	14.143		✕	✕		
	Demodulation		1.24	1.0E+03	1.240	6.200	1.400	14.000	20.200		✕	✕		
	Preamplifier		0.64	2.6E+03	0.246	1.231	1.400	14.000	15.231		✕	✕		
	Voltage offset		0.78	2.2E+04	0.035	0.177	1.400	14.000	14.177		✕	✕		
Drive	VCO	LTC6990	1.90	3.0E+07	0.000	0.000	0.205	1.025	1.025		✕			
	Multiplier	AD835	0.64	3.0E+07	0.000	0.000	16.000	160.000	160.000		✕	✕		
	Offset	LM6154	1.60	3.0E+07	0.000	0.000	1.400	14.000	14.000		✕	✕		
			0.60	1.0E+05	0.006	0.030	1.400	14.000	14.030		✕	✕		
	Bandpass filter		0.57	1.0E+05	0.006	0.029	1.400	14.000	14.029		✕	✕		
	Preamplifier		1.13	1.0E+04	0.113	0.565	1.400	14.000	14.565		✕	✕		
H-V amplifier	-	ISL28291	77.77	6.4E+04	1.215	-	-	-	150.000		✕			
Microcontroller	Microcontroller	SAM4S	-	-	-	-	-	76.500	76.500	✕				
	ADC		-	-	-	-	4.500	14.850	14.850	✕				
	DAC channel 1		2.10	2.0E+03	1.050	2.205	5.000	16.500	18.705	✕				
	DAC channel 2		1.00	1.3E+06	0.001	0.001	5.000	16.500	16.501	✕				
Power supply	+3.3 V	LMR10515	3.30	-	-	76.630	-	-	76.630				881	92
	+5 V	MAX682	5.00	-	-	203.767	-	-	203.767	✕			551	73
	-5 V	LTC1983-5	5.00	-	-	34.865	-	-	34.865		✕		170	83
								**Total:**	958					

Table Supplementary Table 3 | Breakdown by subsystems (corresponding to blocks of Supplementary Figure 2) of the power consumption of the current version of the MEMS system, and the electrical characteristics used for the calculations.

Supplementary Table 3 shows that, except for the wheatstone bridge bias which has the lowest load impedance, the quiescent power dissipation of active integrated circuits (IC) in the analog signal chains (measurement and drive subsystems) is in most cases multiple orders of magnitude larger than their dissipation under load, with signals applied. This points to an obvious optimization step where new components could be selected that are more power efficient in their quiescent state, and their power rails could be reduced from ±5 V to 0-2 V since for given quiescent currents, power dissipation is proportional to the supply voltage (voltage difference between the power rails). The most inefficient devices that could first be optimized are the multiplier IC, the high-voltage amplifier module and the +5 V voltage regulator. The cascaded configuration of the voltage regulators (+3.3V, +5V, -5V, starting from the battery) dissipates unnecessary energy since the (in)efficiency (rightmost column of Supplementary Table 3, taken from the datasheets) of the voltage regulators down the chain gets compounded by the efficiency of each preceding regulator by drawing extra current, increasing the power the latter must output (Supplementary Table 3, penultimate column), and thus their dissipation.

Supplementary Table 4 presents the projected power consumption for the next design iteration of our MEMS system. It was built using the same calculation methodology as the one used for Supplementary

Subsystem	Subcircuit	Active device	V_{out} (V_{rms})	Z_{load} (Ω)	I_{load} (mA_{rms})	P_{load} (mW)	$I_{quiescent}$ (mA_{rms})	$P_{quiescent}$ (mW)	P_{total} (mW)	Improvements
Measurement	Wheatstone bridge	LM4132	2.05	8.0E+02	2.560	3.200	0.060	0.075	3.275	2.048 V reference instead of opamp
	Differential amplifier	MCP6N11	0.88	1.1E+04	0.080	0.100	0.800	1.000	1.100	Operates on lower supply voltage
	Bandpass filter	MAX44280	0.88	4.2E+03	0.210	0.263	0.750	0.938	1.201	Better component
	Demodulation		0.88	1.0E+03	0.884	1.105	0.750	0.938	2.042	Better component
	Preamplifier		0.88	2.6E+03	0.340	0.425	0.750	0.938	1.362	Voltage offset removed
Drive	VCO	LTC6990	2.50	3.0E+07	0.000	0.000	0.205	0.256	0.256	No changes
	Multiplier	MCP4152	-	-	-	-	0.450	0.563	0.563	Design modification
		MAX44280	0.64	1.0E+05	0.006	0.008	0.750	0.938	0.946	Voltage offset removed
	Bandpass filter		0.88	1.0E+05	0.009	0.011	0.750	0.938	0.949	Better component
	Preamplifier		0.88	1.0E+04	0.088	0.110	0.750	0.938	1.048	Better component
H-V amplifier	-	ISL28291	190	-	-	-	-	-	50.000	Circuit redesign for 2.5V operation
Microcontroller	Microcontroller	SAM4L	-	-			4.320	10.800	10.800	Switch to low power microcontroller
	ADC		-	-			0.149	0.372	0.372	
	DAC channel 1		2.10	2.0E+03	1.050	1.313	0.062	0.078	1.391	
	DAC channel 2		1.00	1.3E+06	0.001	0.001	0.062	0.078	0.079	
Power supply	+2.5 V	LT1615	2.50	Power output (mW): 75		Efficiency: 80%			18.846	Switch to 2.5 V
								**Total:**	94	

Table Supplementary Table 4 | Breakdown of the projected power consumption for the next generation of the MEMS system.

Table 3. As hinted in the above paragraph, large improvements in power efficiency could be achieved by using a single +2.5 V supply rail and by changing most active devices to work with this supply, while selecting devices with a high power efficiency and a small form factor. The general circuit architecture would remain the same as that of the current iteration, except for the amplitude modulator which would be implemented using a digitally-controlled variable gain amplifier. These optimizations are expected to provide a reduction of 864 mW, or 90%, over the current design.

After these design modifications, the most important remaining sources of power dissipation will be the high-voltage amplifier with over 50% of the total dissipation, followed by the voltage regulator, the microcontroller and the wheatstone bridge polarization. More complex design modifications could further reduce power consumption. The high voltage driving signal is required to drive the MEMS beam of the current design into a sufficiently nonlinear regime. Adjustments to the beam geometry (such as reducing its width from 4 μm to 1 μm and thickness from 50 μm to 15 μm) could reduce the driving voltage to below 5 V and eliminate the high-voltage amplifier. Other examples of possible design modifications include changing the beam displacement measurement mechanism from piezoresistivity to capacitive transduction, vacuum packaging to increase the quality factor of the mechanical structures, and getting rid of the microcontroller by implementing the delayed feedback loop using a charge-coupled device or by coupling the degrees of freedom of multiple physical oscillators. By removing the contributions to Supplementary Table 4 from the Wheatstone

bridge, the high-voltage amplifier and the microcontroller, total power dissipation falls to 12 mW, with 2.5 mW dissipated by the voltage regulator.

Supplementary References

[1] Erik K. Antonsson and Robert W. Mann. The frequency content of gait. *Journal of Biomechanics*, 18(1):39–47, January 1985.

[2] Guillaume Dion, Salim Mejaouri, and Julien Sylvestre. Reservoir computing with a single delay-coupled non-linear mechanical oscillator. *Journal of Applied Physics*, 124(15):152132, October 2018.

[3] Matthew William Flood, Ben P. F. O'Callaghan, and Madeleine M. Lowery. Gait Event Detection From Accelerometry Using the Teager–Kaiser Energy Operator. *IEEE Transactions on Biomedical Engineering*, 67(3):658–666, March 2020.
