# Supplementary material for: In-sensor human gait analysis with machine learning in a wearable microfabricated accelerometer
Source: Commun Eng. 2024 Mar 16;3:48. doi: 10.1038/s44172-024-00193-5 (PMC10955877; doi:10.1038/s44172-024-00193-5)
Supplement: Supplementary file 3 — Description of Additional Supplementary Files [file 44172_2024_193_MOESM3_ESM.pdf]

# Description of Additional Supplementary Files

**File name:** Supplementary Movie 1

**Description:** Demonstration of the real-time classification of gait patterns with the MEMS wearable device. The MEMS device is attached to the left foot, and it uses in-sensor computing to identify the normal, toe-out and trunk-lean gait patterns with two LED indicators.
